# LmxMPK4, a mitogen-activated protein (MAP) kinase homologue essential for promastigotes and amastigotes of *Leishmania mexicana*

**DOI:** 10.1186/1475-9292-4-6

**Published:** 2005-12-29

**Authors:** Qiong Wang, Inga M Melzer, Martin Kruse, Claudia Sander-Juelch, Martin Wiese

**Affiliations:** 1 Bernhard Nocht Institute for Tropical Medicine, Parasitology Section, Bernhard-Nocht-Strasse 74, D-20359 Hamburg, Germany

## Abstract

**Background:**

*Leishmania *parasites undergo profound morphological and biochemical changes while passing through their life cycle. Protein kinases have been shown to be involved in the differentiation from the extracellular flagellated promastigotes to the intracellular "non-flagellated" amastigotes and vice versa. Moreover, these enzymes are likely involved in the regulation of the proliferation of the different life stages.

**Results:**

Here, we characterize LmxMPK4, a mitogen-activated protein (MAP) kinase homologue from *Leishmania mexicana*. The kinase reveals all sequence motifs for classification as a MAP kinase. LmxMPK4 proved to be active as a recombinant protein. The kinase is expressed in promastigotes and amastigotes. It was impossible to generate homozygous gene deletion mutants for *LmxMPK4 *in promastigotes. Moreover, amastigotes bearing only an episomal copy of the gene stably retained *LmxMPK4 *over a prolonged period without antibiotic pressure in infected mice.

**Conclusion:**

LmxMPK4 is essential for promastigotes and amastigotes of *Leishmania*. It shows significant amino acid sequence divergence to mammalian MAP kinases. Thus, LmxMPK4 is a promising new drug target.

## Background

Protein kinases are key regulatory molecules in all eukaryotic cells. Together with their antagonists the protein phosphatases they form complex networks of mutually activating and silencing molecules. Their activity affects vital processes like differentiation, proliferation, motility, stress response, and apoptosis [1]. Mitogen-activated protein (MAP) kinases are often the final kinases in signal transduction cascades relaying signals augmented by environmental stimuli via multiple steps of reversible phosphorylation from different receptors to effector proteins which can ultimately lead to changes in protein expression profiles. In higher eukaryotes the core of the MAP kinase signal transduction cascade is comprised of a MAP kinase kinase kinase (MKKK), a MAP kinase kinase (MKK) and a MAP kinase. Upon a stimulus the MKKK is activated by phosphorylation. The active MKKK phosphorylates a MAP kinase kinase (MKK) on serine and/or threonine residues in its phosphorylation loop. MKKs are dual-specificity kinases which are able to phosphorylate their substrates, the MAP kinases, on a threonine and a tyrosine residue of the conserved TXY motif of the phosphorylation loop in order to cause maximal enzymatic activity. Finally, activated MAP kinases can translocate into the nucleus and phosphorylate transcription factors or phosphorylate cytosolic kinases or structural proteins. Genome sequencing and analysis of *Leishmania major *and *L. infantum *revealed the presence of all members of the MAP kinase signal transduction core module [2]. However, neither typical receptors that lead to the activation of MAP kinase signal transduction pathways like receptor tyrosine kinases and G-protein coupled receptors nor typical MAP kinase substrates like transcription factors for RNA polymerase II promoters have been found in the parasites. It is not known yet how regulation of gene expression is achieved in the parasites, but it is likely that the substrates of MAP kinases are gene regulatory molecules, albeit not transcription factors. It is generally assumed that regulation occurs on post-transcriptional levels like the maturation of the mRNA from a polycistronic precursor to a capped and poly-adenylated mRNA in trans splicing, the mRNA stability, the efficiency of translation or even the protein stability.

*Leishmania *parasites have a digenetic life cycle with mammals and sand flies as their hosts. While passing through their life cycle the parasites alternate between proliferative forms, the procyclic promastigotes and the amastigotes, and forms arrested in the cell cycle, the highly infectious metacyclic promastigotes. Protein kinases are likely involved in the regulation of the proliferation of the different life stages and in the differentiation processes from procyclic promastigotes to metacyclic forms, to amastigotes after transmission to a mammal and uptake by host macrophages, and back to promastigotes once the amastigotes reach the gut of a sand fly. Members of the MAP kinase signalling pathways have been found to play important roles in differentiation and proliferation of *Leishmania*. LmxMKK a MAP kinase kinase homologue is involved in the formation and maintenance of the flagellum in the promastigote. A null mutant displayed flagella reduced to maximally 1/5 of the length of the wild type flagellum [3]. It is likely that this kinase is involved in the outgrowth of a new flagellum prior to cell division in the promastigotes and in the differentiation from the amastigote displaying a short flagellum not protruding from the flagellar pocket, an invagination of the plasma membrane at the base of the flagellum, to the promastigote with a flagellum which could even be longer than the body of the cell. With LmxMPK9 a second kinase belonging to a MAP kinase signalling pathway has been found which is involved in the regulation of flagellar length. Here, null mutant promastigotes showed elongated flagella as compared to wild type cells, indicating that this kinase affects the shortening of the flagellum [4]. LmxMPK1, the first MAP kinase homologue described in *L. mexicana*, has been found to be essential for the proliferation of the amastigotes [5]. A deletion mutant was still able to infect host cells, transform to the amastigote morphology, but was unable to proliferate. As such, this kinase is an ideal drug target and a specific inhibitor would be promising to treat the disease.

Here, we characterize LmxMPK4, a MAP kinase homologue from *L. mexicana*, which is essential for promastigotes and amastigotes, and consequently also has the potential to be used as a drug target to treat Leishmaniasis.

## Results

### Molecular characterisation of LmxMPK4

**Figure 1:**
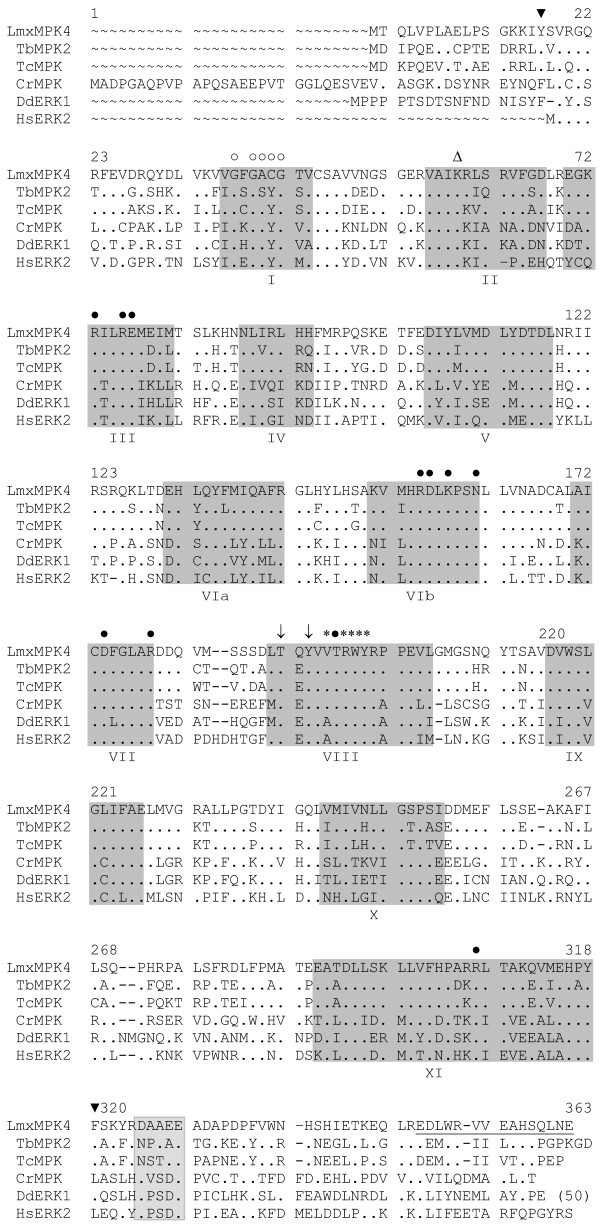
**Alignment of LmxMPK4 from *L. mexicana *with various MAP kinase amino acid sequences**. LmxMPK4, *Leishmania mexicana *MAP kinase 4 (GenBank accession number AJ293282; [6]); *Trypanosoma brucei *MAP kinase, TbMAPK2 (GeneDB accession number Tb10.70.2070; [10]); *Trypanosoma cruzi *MAP kinase (GeneDB accession number Tc00.1047053511299.70); *Chlamydomonas reinhardtii *MAP kinase (GenBank accession number BAB18271); *Dictyostelium discoideum *ERK1 (GenBank accession number U11077; [13]); *Homo sapiens *ERK2 (GenBank accession number CAA77753; [14]). Roman numerals I to XI indicate kinase subdomains. The kinase domain is located between the two inverted triangles (▼). Arrows (↓) mark the potential regulatory phosphorylation sites in the TXY motif at Thr190 and Tyr192, filled circles (●) indicate conserved amino acid residues it is not clear what you mean by conserved aa residues, and open circles (○) depict the residues of the phosphate anchor ribbon for ATP binding. The asterisks (*) show the residues of the P+1 specificity pocket. The open triangle (Δ) marks the invariant lysine residue (K59), which is essential for the phosphate transfer reaction. The CD-domain is shaded in light grey. Dashes indicate gaps introduced to optimize the alignment; dots represent identical residues. The carboxy-terminal peptide used to generate antibodies is underlined. (50), the terminal 50 amino acid residues of the *Dictyostelium *kinase have been omitted. Numbering corresponds to LmxMPK4.

**Figure 2:**
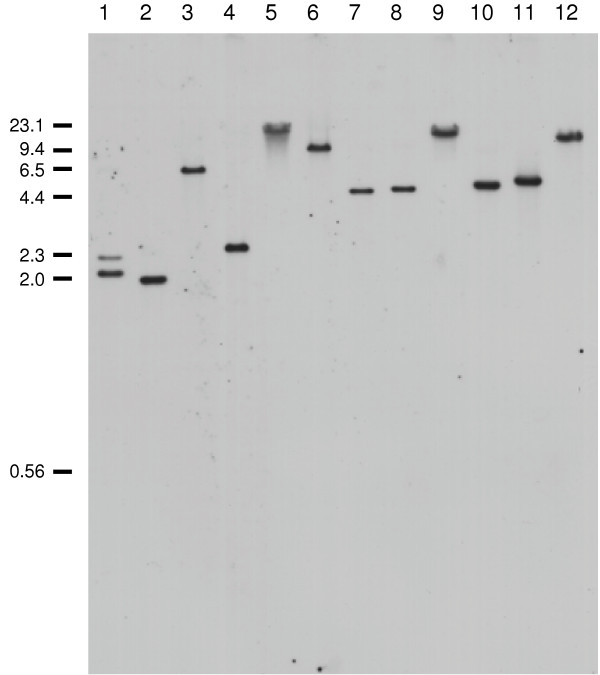
**Southern analysis of genomic DNA**. 1, *Pst*I; 2, *Hin*cII; 3, *Hin*dIII; 4, *Acc*65I; 5, *Xba*I; 6, *Spe*I; 7, *Eco*RV; 8, *Cla*I; 9, *Nde*I; 10, *Nco*I; 11, *Mlu*I; 12, *Mun*I. Digested DNA (5 μg) was electrophoresed on a 0.7% agarose gel, blotted onto nylon membranes and probed with a DIG-labelled DNA probe corresponding to a fragment of the open reading frame (nt 240 – nt 930). Numbers indicate the approximate size of DNA markers in kb.

We cloned and sequenced the gene for LmxMPK4, which is a MAP kinase homologue of *L. mexicana*. The open reading frame (orf) of LmxMPK4 is comprised of 1089 bp coding for a protein of 363 amino acids and a calculated molecular mass of 41.5 kDa [6]. LmxMPK4 is highly conserved in the different *Leishmania *species (*L. panamensis *98.6%, *L. donovani *97.5%, *L. infantum *97.5%, and *L. major *98.9% amino acid identities) and has homologues in other kinetoplastids, like *Trypanosoma brucei *(67.2%; E value of 5e^-139^) and *T. cruzi *(67.4%; E value of 2e^-143^), in *Chlamydomonas rheinhardtii *(43.9%; E value of 6e^-82^), and in *Dictyostelium discoideum *(45.0%; E value of 1e^-85^). Moreover, it shows weak homology to the human MAP kinases ERK1 (42%; E value of 1e^-70^) and ERK2 (42%; E value of 6e^-71^). LmxMPK4 contains the twelve kinase subdomains and amino acid residues known to be highly conserved in MAP kinases (Fig. 1). The kinase domain reaches from tyrosine 17 to phenylalanine 318 encompassing nearly the entire protein leaving only a sixteen residue amino-terminal and a 45 residue carboxy-terminal region. The latter region reveals a potential common docking (CD) domain (DAAEE), a region enriched for negatively charged residues involved in the binding of interacting proteins [7]. Subdomain I contains the phosphate anchor ribbon for ATP binding with the consensus (GXGXXG). A conserved lysine residue (K59), which is involved in orienting ATP for proper phosphate transfer is located in subdomain II. The TXY motif carrying a glutamine in its centre in LmxMPK4, and the P+1 specificity pocket, both hallmarks for MAP kinases are present in subdomain VIII. Finally, a number of other conserved residues are found in the different subdomains. Southern blot analysis of genomic DNA from *L. mexicana *showed that LmxMPK4 is a single copy gene in the haploid genome (Fig. 2). *Pst*I cuts within the DNA fragment used as a probe and thus showed two hybridizing bands whereas all other restriction endonucleases only revealed a single band.

### Immunoblot analysis

**Figure 3:**
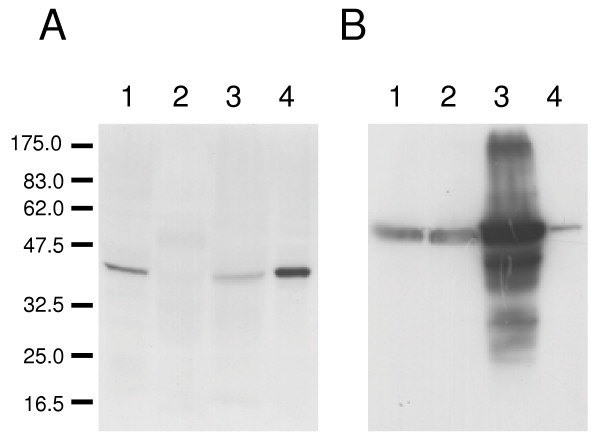
**Immunoblot of LmxMPK4 from *L. mexicana *wild type and episomal overexpressor**. Lane 1, *L. mexicana *wild type promastigotes (4 × 10^7 ^cells); lane 2, amastigotes derived from mouse lesion (4 × 10^7 ^cells); lane 3, wild type *in vitro *differentiated amastigotes (1 × 10^8 ^cells); lane 4, promastigotes of LmxMPK4 episomal overexpressor (1 × 10^7 ^cells). A, blot probed with antiserum against the carboxy-terminal peptide of LmxMPK4; B, the blot was stripped and re-probed with a polyclonal antiserum against myo-inositol-1-phosphate synthase. The molecular masses of standard proteins are indicated in kilodaltons.

Using a polyclonal antiserum raised in rabbits against a carboxy-terminal peptide of LmxMPK4, the protein could be detected in a lysate of 4 × 10^7 ^wild type promastigotes after lipid extraction, but was hardly detectable in the same number of lesion-derived amastigotes (Fig. 3, lanes 1 and 2). However, we could detect it in *in vitro *differentiated and cultivated amastigotes which had been under amastigote growth conditions for ten passages. Even here 1 × 10^8 ^cells had to be extracted and loaded onto the gel to generate a faint band indicating that the protein is down-regulated in the amastigotes (Fig. 3A, lane 3). On the other hand, we could easily overexpress LmxMPK4 from an episome in promastigotes leading to an at least tenfold overexpression (Fig. 3A, lane 4). To demonstrate differential loading of protein amounts the blot was stripped and re-probed with an antiserum against myo-inositol-1-phosphate synthase, a protein known to be equally expressed in promastigotes and amastigotes [8].

### Expression of recombinant LmxMPK4 and kinase assay

**Figure 4:**
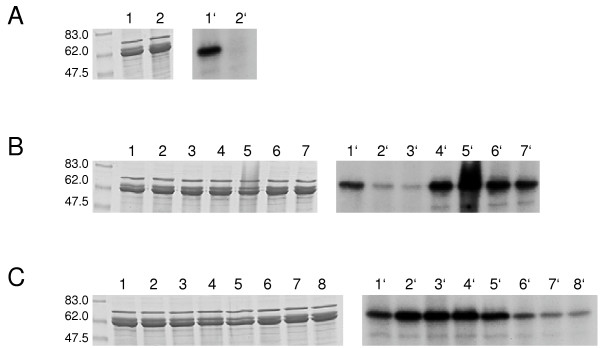
**Recombinant expression of LmxMPK4 and kinase assay**. Left panels, Coomassie-stained gels; right panels, autoradiographs. A, recombinant GST-fusion proteins after kinase assay. Lanes 1 and 1', wild type LmxMPK4; lane 2 and 2', KM-mutant of LmxMPK4. B, kinase assay of GST-LmxMPK4 at different concentrations of Mg^2+ ^and Mn^2+^. Lanes 1 and 1', 50 mM Mg^2+^; lanes 2 and 2', 10 mM Mg^2+^; lanes 3 and 3', 5 mM Mg^2+^; lanes 4 and 4', 10 mM Mg^2+ ^and 2 mM Mn^2+^; lanes 5 and 5', 10 mM Mn^2+^; lanes 6 and 6', 2 mM Mn^2+^; lanes 7 and 7', 1 mM Mn^2+^. C, kinase assay of GST-LmxMPK4 at varying pH. Lanes 1 and 1', pH5.5; lanes 2 and 2', pH6.0; lanes 3 and 3', pH6.5; lanes 4 and 4', pH7.0; lanes 5 and 5', pH7.2; lanes 6 and 6', pH7.5; lanes 7 and 7', pH8.0; lanes 8 and 8', pH 8.5. Molecular masses of standard proteins are indicated in kilodalton.

To generate an enzymatically inactive KM-mutant LmxMPK4 was mutated changing lysine 59 to methionine. Both, the wild type and the mutant protein, were subcloned into pGEX-KG [9] and expressed as recombinant glutathione S-transferase fusion proteins in *Escherichia coli*. Both proteins resulted in the same pattern of bands after enrichment on glutathione-sepharose, separation on SDS-polyacrylamide gels and staining with Coomassie brilliant blue. The main contaminating bands were the glutathione S-transferase fusion partner (not shown), a less abundant protein of a higher molecular mass, and a second band just below the GSTLmxMPK4 fusion protein (Fig. 4A, lanes 1 and 2). The latter contaminations were also found while expressing unrelated MAP kinases from *L. mexicana *in *E. coli *and therefore are likely of bacterial origin (data not shown). In kinase assays we could detect autophosphorylation of LmxMPK4, but not of the KM-mutant (Fig. 4A, lanes 1' and 2') indicating that the phosphorylation of LmxMPK4 is not due to a bacterial kinase which might have been co-purified. However, the kinase activity was relatively weak as films had to be exposed for up to three days to obtain the results shown in figure 4. Phosphorylation of myelin basic protein (MBP) could be obtained with some preparations of recombinant LmxMPK4, but not others (data not shown). We usually perform kinase assays in the presence of 10 mM Mg^2+ ^and 2 mM Mn^2+ ^at 27°C. It turned out that manganese on its own in a concentration of 10 mM worked best in kinase assays for LmxMPK4 (Fig. 4B). Finally, a pH between 6.0 and 7.0 showed highest autophosphorylation activity in the assay (Fig. 4C).

### Deletion analysis of LmxMPK4

**Figure 5:**
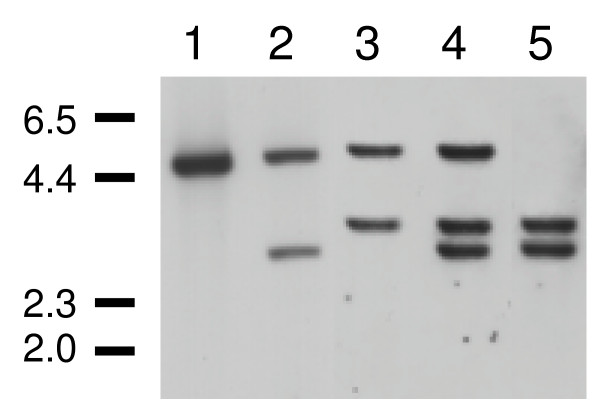
**Deletion analysis of LmxMPK4**. Southern blot analysis of different clones with a probe corresponding to the 3'-UTR of *LmxMPK4*. 1, *L. mexicana *wild type; 2, episome pXPACLmxMPK4 + Δ*LmxMPK4*+/- Phleo; 3, episome pXPACLmxMPK4 + Δ*LmxMPK4*+/- Hyg; 4, episome pXPACLmxMPK4 + Δ*LmxMPK4*-/-/+ Phleo/Hyg; 5, episome pXPACLmxMPK4 + Δ*LmxMPK4*-/- Phleo/Hyg. Numbers indicate the approximate size of DNA markers in kb.

To investigate the function of LmxMPK4 in *Leishmania *parasites we attempted to obtain homozygous deletion mutants. In order to delete both alleles of the single copy gene we generated constructs comprised of the 5'- and 3'-untranslated regions (UTRs) of *LmxMPK4 *flanking the hygromycin B phosphotransferase gene or the gene of the phleomycin binding protein. Following first rounds of homologous recombination we obtained single-allele deletion mutants for all constructs (data not shown). All our attempts to generate a null mutant for *LmxMPK4 *resulted in clones carrying the resistance marker genes in the expected genomic context, however, they all retained an extra copy of *LmxMPK4 *(data not shown). This result seemed to be indicative for a gene which is required for the promastigote life stage of *L. mexicana*. Therefore, we decided to first add an episomal copy of the *LmxMPK4 *wild type gene into the promastigotes using the plasmid pXPACLmxMPK4. The presence of the plasmid and the degree of expression of the MAP kinase was checked by Southern and immunoblot analyses (data not shown; Fig. 2, lane 4). Next we repeated our deletion strategy, this time leading to a successful replacement of the genomic alleles of *LmxMPK4 *in one clone (Fig. 5, lane 5), whereas most of the recombinant parasites retained a genomic allele of LmxMPK4 despite the presence of the gene on the episome (an example is shown in Fig. 5, lane 4). The resulting genomic null mutant parasites showed no obvious phenotype compared to the wild type promastigotes. We used this cell line to infect female Balb/c mice and monitored lesion development for more than 1.5 years. All mice injected showed lesion development (data not shown). Towards the end of the experiment we either sacrificed the mice and isolated the lesion-derived amastigotes for transformation to promastigotes, or took aspirates of the lesion for polymerase chain reaction (PCR) analysis to assess the absence or presence of the plasmid carrying *LmxMPK4*. PCR on the total population of freshly differentiated promastigotes proved the presence of *LmxMPK4 *in the population of cells. Re-isolation of plasmids from freshly differentiated promastigotes and restriction analysis confirmed the identity of the plasmid (data not shown). To evaluate the presence of plasmids in amastigotes we isolated parasites by taking lesion aspirates. As a control wild type amastigotes were isolated and treated like the mutant amastigotes. The amastigotes were liberated from their host cells by shear forces also disrupting aggregates of amastigotes leading to a suspension of single cells. The DNA of the cells was stained using SYTO16 (Invitrogen, Heidelberg, Germany) followed by sorting of the parasites into 96-well plates. A duplex PCR was performed using a pair of oligonucleotide primers specific for the single-copy gene *LmxMPK9*, which generates a 254 bp DNA fragment, and a second pair of oligonucleotides specific to detect the presence of *LmxMPK4 *by a 420 bp DNA fragment. In a series of PCRs using different numbers of sorted wild type amastigotes it turned out that at least ten amastigotes per well had to be used to give reliable results in the subsequent PCR. Only one reaction out of 44 failed to show the control fragment for *LmxMPK9 *in the mutant. This rare event of no amplification was not found for *LmxMPK9 *in the wild type but was observed for *LmxMPK4 *in both wild type and mutant amastigotes indicating that it is indeed a PCR failure and the plasmid is definitely present in all cells. Looking at freshly differentiated promastigotes we obtained a significantly different picture. As promastigotes could be purified much easier we succeeded to perform the duplex PCR on the single cell level with high efficiency. Again a failure of the PCR could be observed, as in 5% of all wells (4/77) the control fragment was not amplified. The percentage of wells lacking the *LmxMPK4 *fragment was 27% (21/77). This higher percentage is indicative for the presence of single cells lacking the plasmid and thus the gene. As this gives no clue about the ability of those promastigotes having lost the plasmid to proliferate, we cloned the cells by serial dilution and analysed the populations arising from single cells using the duplex PCR. All 66 clones obtained still retained the plasmid. Therefore, LmxMPK4 is essential for promastigotes and amastigotes.

## Discussion

LmxMPK4 has been found to be a MAP kinase homologue from *L. mexicana *by amino acid sequence comparison and an active protein kinase by proving the activity of the recombinant GST-fusion protein in *in vitro *kinase assays. The protein was hardly detectable in amastigotes by immunoblot analysis, however, the fact that amastigotes lacking the genomic copies of *LmxMPK4 *retained a plasmid carrying the gene without any antibiotic selection imposed on the cells while in the mouse for more than 1.5 years strongly suggests that the protein is expressed and essential for this life stage. Moreover, all attempts to generate homozygous deletion mutants in the promastigotes failed and constantly ended in parasites showing accurate replacement of both alleles of *LmxMPK4*, but an additional copy of the gene which might reflect a chromosomal duplication. Thus, LmxMPK4 is required in the insect stage promastigotes as well. This is contrary to TbMAPK2, the *T. brucei *homologue of LmxMPK4, which could be deleted in the mammalian stage bloodstream form trypanosomes without a detectable phenotype [10]. However, differentiation of the null mutant to procyclic trypanosomes by temperature shift from 37°C to 27°C and addition of cis-aconitate revealed delayed differentiation kinetics and an arrest of the parasites in all phases of the cell cycle. It is likely, that in *T. brucei *procyclic forms cell cycle progression depends on a constitutive stimulus through TbMAPK2 signalling. In *Leishmania *the importance of LmxMPK4 is even extended to the mammalian stage of the parasite making this kinase a potential drug target to treat Leishmaniasis.

## Conclusion

LmxMPK4 is a mitogen-activated protein kinase homologue from *L. mexicana*, that is highly conserved throughout different *Leishmania *species and is essential for the two main proliferating life stages, the promastigotes and amastigotes, of the parasite. Moreover, it reveals significant amino acid divergence compared to MAP kinases from mammals showing highest homology to protein kinases from kinetoplastids, low homology to human kinases and kinases from *Chlamydomonas *and *Dictyostelium*. Therefore, it represents a suitable and promising drug target to prevent the proliferation of the parasites and cure the disease.

## Methods

### Parasites

Promastigotes of *L. mexicana *MNYC/BZ/62/M379 were grown as described [11]. Female Balb/c mice were infected into the left hind foot pad at the age of 6–8 weeks with 1 × 10^7 ^promastigote parasites in 30 μl PBS (137 mM NaCl, 2.7 mM KCl, 8 mM Na_2_HPO_4_, 1.4 mM KH_2_PO_4_). Infection experiments have been approved by the Veterinary Authorities of the Freie and Hansestadt Hamburg and were performed in accordance with section 8a paragraph 1 together with section 10a of the German Animal Protection Law as amended on May 25, 1998, with latest amendment from August 6, 2002 (GZ.: G 21132/591-00.33). Using a caliper gage, the course of infection was followed by measuring lesion size relative to the uninfected right hind footpad at 4–5 weeks intervals. Amastigotes were isolated from lesions of Balb/c mice as described [5]. For small scale amastigote preparation the lesion was aspirated 2–3 times with a 23 G × 1 1/4" needle and a syringe. The isolated tissue was transferred into 0.5 ml of cold SYTO16-buffer (21 mM Hepes pH 7.5, 137 mM NaCl, 5 mM KCl, 6 mM Glucose) and kept on ice. The tissue was passed five times through a 30 G × 1/2" needle to generate single cells. 850 μl of cold SYTO16-buffer, 150 μl DMSO and 0.5 μl of 33 μM SYTO16 (Invitrogen) in DMSO were added, the cells mixed and incubated for 30 minutes at room temperature in the dark. Afterwards the cells were washed twice with SYTO16-buffer and were finally resuspended in 1 ml SYTO16-buffer and stored at 4°C in the dark before sorting. For promastigote staining 1.1 × 10^8 ^cells were taken from a log-phase culture, washed twice with 1 ml SYTO16-buffer, and processed as described above.

### Gene cloning, sequencing and analysis

Positive phage clones for *LmxMPK4 *were selected using digoxigenin (DIG)-labelled probes [6], amplified and DNA inserts cloned into pBSKII(+) using *Eco*RI (Stratagene, La Jolla, CA) yielding pBE25LmxMPK4. A 4514 bp *Cla*I DNA fragment was liberated from pBE25LmxMPK4 and ligated into pBSKII(+) yielding pB2CLmxMPK4. Both DNA strands were sequenced. Plasmid isolation, DNA sequencing and analysis, DNA isolation and blotting, and hybridizations were performed basically as described before [12]. A probe encompassing nt 204 – nt 930 of the orf of *LmxMPK4 *was DIG-labelled using the oligonucleotides 5'-TGCGGGAGATGGAGATAA-3' and 5'-CTTCGCAGTTAATCGTCTT-3' and the PCR DIG probe synthesis kit as described by the manufacturer (Roche, Mannheim, Germany). For labelling of the 2350 bp *Sph*I/*Nru*I DNA fragment corresponding to the 3' untranslated region of *LmxMPK4 *the fragment was isolated from pB2CLmxMPK4 and labelled using the DIG DNA labelling kit (Roche). *LmxMPK4 *sequence data have been submitted to the DDBJ/EMBL/GenBank databases under the accession number AJ293282.

### *LmxMPK4 *deletion constructs

The Expand™ High Fidelity PCR or the Expand Long Template PCR System (Roche) was used for all polymerase chain reaction (PCR) applications. To generate the construct for a genomic allele deletion mutant for *LmxMPK4 *the flanking regions were amplified by PCR from pB2CLmxMPK4 cut with *Bgl*II using 5'-GCTGAGCCATGGTTCTCTATGCCTCC-3' and 5'-TGAAAGCCTAGGAATCTGGTGCTCTC-3' (3 min 94°C, 10 × [20 sec 94°C, 30 sec 45°C, 7 min 68°C], 20 × [20 sec 94°C, 30 sec 45°C, 7 min + 20 sec/cycle 68°C], 7 min 68°C, 4°C). The gel-purified PCR-fragment was trimmed at the ends using *Nco*I and *Avr*II, and ligated to a *Bsp*HI/*Nhe*I fragment containing either the phleomycin binding protein gene (*BLE*) or the hygromycin B phosphotransferase gene (*HYG*) as described before [12], yielding pB11CΔLmxMPK4phleo and pB21CΔLmxMPK4hyg. Both constructs were cut with *Cla*I and *Nru*I, the DNA fragments for homologous recombination were gel-purified and used for electroporation in two consecutive rounds and recombinants selected in medium containing 5 μg ml^-1 ^bleocin and 20 μg ml^-1 ^hygromycin B [4].

### Episomal expression constructs for LmxMPK4

For episomal expression of *LmxMPK4 *the orf was amplified from pB2CLmxMPK4 using 5'-CCCGATA**TC****ATGA**CTCAGCTCGTCCC-3' and 5'-CCCGATAT**C****TCGAG**CCTATTCGTTCAATTGTG-3' (5 min 94°C, 20 × [30 sec 94°C, 30 sec 45°C, 1 min 72°C], 7 min 72°C, 4°C) introducing *Eco*RV, *Bsp*HI, and *Xho*I restriction sites, trimmed with *Eco*RV and ligated into pBSKII(+) to yield pB6ELmxMPK4. Both DNA strands were sequenced. The 1104 bp *Eco*RV fragment carrying *LmxMPK4 *was liberated and cloned into pX63polPAC linearised at its single *Eco*RV site [4]. Cells were transfected with 20 μg of the plasmid constructs designated pXPACLmxMPK4 as described above, and transformants were selected in SDM-79 using 20 μM puromycin.

### Expression constructs, mutagenesis and antibody production

The 1095 bp *Bsp*HI/*Xho*I DNA fragment was isolated from pB6ELmxMPK4, ligated into pGEX-KG [9] cut with *Nco*I and *Xho*I, and the resulting construct was transformed into *E. coli *XL1-blue. Expression and purification of the glutathione-S-transferase fusion protein was performed as described before [4]. To generate an enzymatically inactive version of LmxMPK4 lysine 59 was replaced by methionine. The oligonucleotides 5'-GTTG**TCGCGA**GTCTTTGGTGATCTTCGTG-3' and 5'-AGAC**TCGCGA**CAACCGCATGATAGCCACTCGCTCACC-3' introducing *Nru*I sites and the desired mutation were used in a PCR on pB6ELmxMPK4 (94°C, 5 min; 10 × [94°C, 30 s; 45°C, 30 s; 68°C, 3 min]; 10 × [94°C, 30 s; 55°C, 30 s; 68°C, 3 min + 20 s/cycle]; 68°C, 7 min; 4°C). The amplified fragment was gel-purified, trimmed at the ends using *Nru*I, circularised by self-ligation, and transformed into *E. coli*. Successful mutagenesis was confirmed by sequencing. Finally, the mutated gene was cloned into pGEX-KG as described above. A rabbit antiserum was produced against the peptide CEDLWRVVEAHSQLNE corresponding to the 15 carboxy-terminal amino acids of LmxMPK4 (Eurogentec, Seraing, Belgium).

### Immunoblotting

Cells were harvested by centrifugation and washed once using PBS. The pellet was resuspended in chloroform/methanol/ddH_2_O (1:2:0.8) in a concentration of 2 × 10^8 ^cells/ml and left at room temperature for 30 min. The extracted cells were pelleted for 10 min at 15000 × g at room temperature and the pellet dried under vacuum (speed-vac). Then the pellet was resuspended in 100 μl 50 mM Tris/HCl pH 8.0, 20 μM leupeptin, 1 mM phenylmethylsulfonyl fluoride, 5 mM 1,10-phenanthroline, 10 mM MgCl_2_, 100 U/ml benzonase (Merck, Darmstadt, Germany) and incubated at 37°C for 30 min. Dithiothreitol (DTT) was added to a final concentration of 50 mM and the probe adjusted to 1 × SDS sample buffer (0.4% SDS, 4% glycerol, 0.0002% bromphenol blue, 50 mM DTT, 12.5 mM Tris-HCl pH 6.8). Probes were boiled for 10 min, 20–40 μl subjected to SDS-polyacrylamide gel electrophoresis (PAGE) and blotted to poly(vinylidenedifluoride) (PVDF) membranes. Immunodetection was carried out as described before [5] using the polyclonal rabbit antiserum and goat-anti-rabbit secondary antibodies coupled to peroxidase (Dianova, Hamburg, Germany) followed by chemiluminescence using the ECL system (Amersham Pharmacia Biotech, Freiburg, Germany).

### Kinase assay

1 μg of the purified kinase was used in 50 μl kinase assay solution (50 mM Tris pH 7.5, 0–50 mM MgCl_2_, 0–10 mM MnCl_2_, 0.1 M NaCl, 5 μCi (γ-^32^P)-ATP, 1 mM ATP, 5 μg myelin basic protein) and incubated at 27°C. The different pH conditions tested were reached using 50 mM morpholinoethane sulfonic acid for pH6.5 and below, 3-(N-morpholino)propanesulfonic acid for pH7 and 7.2, and 50 mM Tris/HCl for pH 7.5 and higher. 12.5 μl 5 × SDS sample buffer were added and the solution heated for 10 min at 95°C. 25 μl of the solution were separated on a 12% SDS-PAGE, stained with Coomassie brilliant blue, destained, dried and exposed to X-ray films at -70°C.

### Cell sorting and PCR analysis

Single promastigotes stained with SYTO16 were gated with a forward scatter FSC-A < 80 and a side scatter SSC-A < 80 and sorted into skirted 96-well PCR plates (Sarstedt, Nuembrecht, Germany) using a FACSAria (Becton & Dickinson, Franklin Lakes, NJ). For lesion-derived amastigotes the smallest SYTO16-positive particles (FSC-A < 50, SSC-A < 50) were sorted with ten cells per well. All PCR reactions were performed in an Eppendorf Mastercycler ep (Eppendorf, Hamburg, Germany). 25 μl of a mastermix were added to each well that contained pro- or amastigotes. 24 μl of mastermix and 1 μl of genomic DNA (c = 0.24 μg/μl) of *L. mexicana *served as a positive control, as a negative control 25 μl of mastermix were used. After addition of the mastermix the plates were sealed with adhesive foil (Sarstedt). 0.5 μl AccuPrime *Taq *DNA Polymerase (Invitrogen) were used in a volume of 25 μl with 5 pmol of the respective oligonucleotide primers (10 min 94°C, 45 × [10 sec 94°C, 15 sec 60°C, 45 sec 68°C], 7 min 68°C, 4°C). For the amplification of the 254 bp control DNA fragment derived from *LmxMPK9 *[6] we used 5'-GTCAGCGTGCCAATGAAAT-3' and 5'-CAAGCTCCGGTGCGCGGTA-3'. A 420 bp DNA fragment corresponding to *LmxMPK4 *is amplified by 5'-TATGATTCAAGCATTCCGCG-3' and 5'-CGGCCGATGCGGCTGAGAG-3'. The samples were analysed on 1.5% agarose gels.

## Competing interests

The author(s) declare that they have no competing interests.

## Authors' contributions

Q.W. cloned and sequenced LmxMPK4, constructed the deletion and overexpression constructs, and performed the deletion analysis and immunoblotting experiments.

I.M.M. expressed and purified the recombinant proteins and performed the enzymatic characterisation.

M.K. isolated amastigotes, prepared cells for FACS sorting and performed PCR analysis on sorted cells.

C.S.-J. performed the cell sorting on a Becton Dickinson FACSAria.

M.W. conceived the study, supervised the execution, did PCR analysis on cloned cell lines, and prepared the final draft of the manuscript.

All authors read and approved the final manuscript.
